# The complete plastome of *Syzygium odoratum* (Lour.) DC. 1928 (Myrtaceae)

**DOI:** 10.1080/23802359.2022.2067015

**Published:** 2022-04-25

**Authors:** Li-Da Chen, Hua-Feng Wang, De-Jia Hou

**Affiliations:** a Sanya Nanfan Research Institute of Hainan University, Hainan Yazhou Bay Seed Laboratory, Sanya, China; b College of Sciences, Huazhong Agricultural University, Wuhan, China

**Keywords:** Genome structure, plastome, *Syzygium*, *Syzygium odoratum* (Lour.) DC. 1828

## Abstract

*Syzygium odoratum* (Lour.) DC. 1828 is a deciduous shrub in the family Myrtaceae. This species grows in sparse forests, especially in mountains, valleys, and broad-leaved evergreen forests along streams from 100 to 400 m above sea level. The primary distribution is in southern China (e.g. Guangdong, Guangxi, Hainan, etc.) and other south Asian countries (e.g. Vietnam). Here, we report and characterize the complete plastome from a cultivar of *S. odoratum* (Lour.) DC. 1828. The complete plastome is 159,352 bp in length with a typical structure and gene content found in angiosperms, including two inverted repeat regions (IRs) of 26,472 bp, a large single-copy (LSC) region of 87,993 bp, and a small single-copy (SSC) region of 18,415 bp. The plastome contains 132 genes, consisting of 84 protein-coding genes, 37 tRNA genes, and eight rRNA genes. The overall G/C content in the plastome of *S. odoratum* is 36.9%. By inferring phylogenetic relationships based on the existing data of related taxa, we find that *S. odoratum* is most closely related to *Syzygium acuminatissimum*, (Blume) DC. 1828 given the current sampling. The complete plastome sequence of *S. odoratum* will provide a useful resource for conservation genetics of this species, as well as for phylogenetic studies involving Myrtaceae.

*Syzygium odoratum* (Lour.) DC. 1828 is a deciduous shrub in the family Myrtaceae. This species grows in sparse forests, especially in mountains, valleys, and broad-leaved evergreen forests along streams from 100 to 400 m above sea level. The primary distribution is in southern China (e.g. Guangdong, Guangxi, Hainan, etc.) and other south Asian countries (e.g. Vietnam). *Syzygium odoratum* can be used as materials for vehicles, sleepers, bridges, and ship building. Additional important ecological functions include acting as strong windbreaks and sand fixation, therefore, the species can be used for coastal sand greening (Chen 1984). We report the complete plastome of *S. odoratum* in this study (GenBank accession number is MZ929420.1), to improve the quality of collections, medicinal applications, and phylogenetic investigations focused on Myrtaceae.

In this study, fresh leaves of *S. odoratum* were sampled from Ledong county in Hainan, China (109.17°E, 18.75°N). The leaves were dried in silica gel. A voucher specimen (voucher code: Wang et al., A254) and its associated DNA have been deposited in the Herbarium of the Institute of Herbarium of China National GenBank (code of herbarium: HUTB) at Hainan University (https://ha.hainanu.edu.cn/home2020/, H.-F. Wang and hfwang@hainanu.edu.cn) under the voucher number Wang et al. A254. Total genomic DNA was extracted from dried leaf tissue with the cetyltrimethyl ammonium bromide (CTAB) protocol of Doyle and Doyle (1987).

This experiment was reported by Zhu et al. (2018) with the sequencing having been done on the BGISeq-500 platform. Cleaned sequencing data of *S. odoratum* were used for plastome assembly using MITObim v1.8 (Hahn et al. 2013). Geneious R8.0.2 (Biomatters Ltd., Auckland, New Zealand) was used to annotate the plastid assembly (MZ929420.1) and the annotation was corrected with DOGMA (Wyman et al. 2004). The results of our study indicate that the complete length of the plastome of *S. odoratum* encompasses 159, 352 bp with the typical quadripartite structure of angiosperms, including two inverted repeats (IRs) of 26,472 bp, a large single-copy (LSC) region of 87,993 bp, and a small single-copy (SSC) region of 18,415 bp. The plastome contains 132 genes, consisting of 84 protein-coding genes (seven of which are duplicated in the IR), 37 tRNA genes (eight of which are duplicated in the IR), and four rRNA genes (5 S rRNA, 4.5 S rRNA, 23 S rRNA, and 16 S rRNA; all are duplicated in the IR). The overall G/C content of the *S. odoratum* plastome is 36.9%, with the corresponding values of the LSC, SSC, and IR region being 34.7%, 30.6%, and 42.7%, respectively. Comparison of the *S. odoratum* plastome to previously published data shows a high level of gene synteny with one publicly available species, *Syzygium acuminatissimum* (Blume) DC. 1828.

We used RAxML (Stamatakis 2006) with 1000 bootstraps under the GTRGAMMAI substitution model to reconstruct a maximum-likelihood (ML) phylogeny of eight published complete plastomes of *Syzygium*, using *Qualea grandiflora* Mart. 1824, (NC043803.1) and *Salvertia convallariodora* A. St.-Hil. 1820 (NC043806.1) as outgroups. By inferring phylogenetic relationships based on the existing data of related taxa, we find that *S. odoratum* is most closely related to *S. acuminatissimum* given the current sampling (Figure 1). Most nodes in the plastome ML tree were highly supported. The plastid sequence of *S. odoratum* has promotes the relevant conservation and phylogenetic investigation of Myrtaceae, bringing great benefits to deepen the understanding of species within the family.

**Figure 1.**
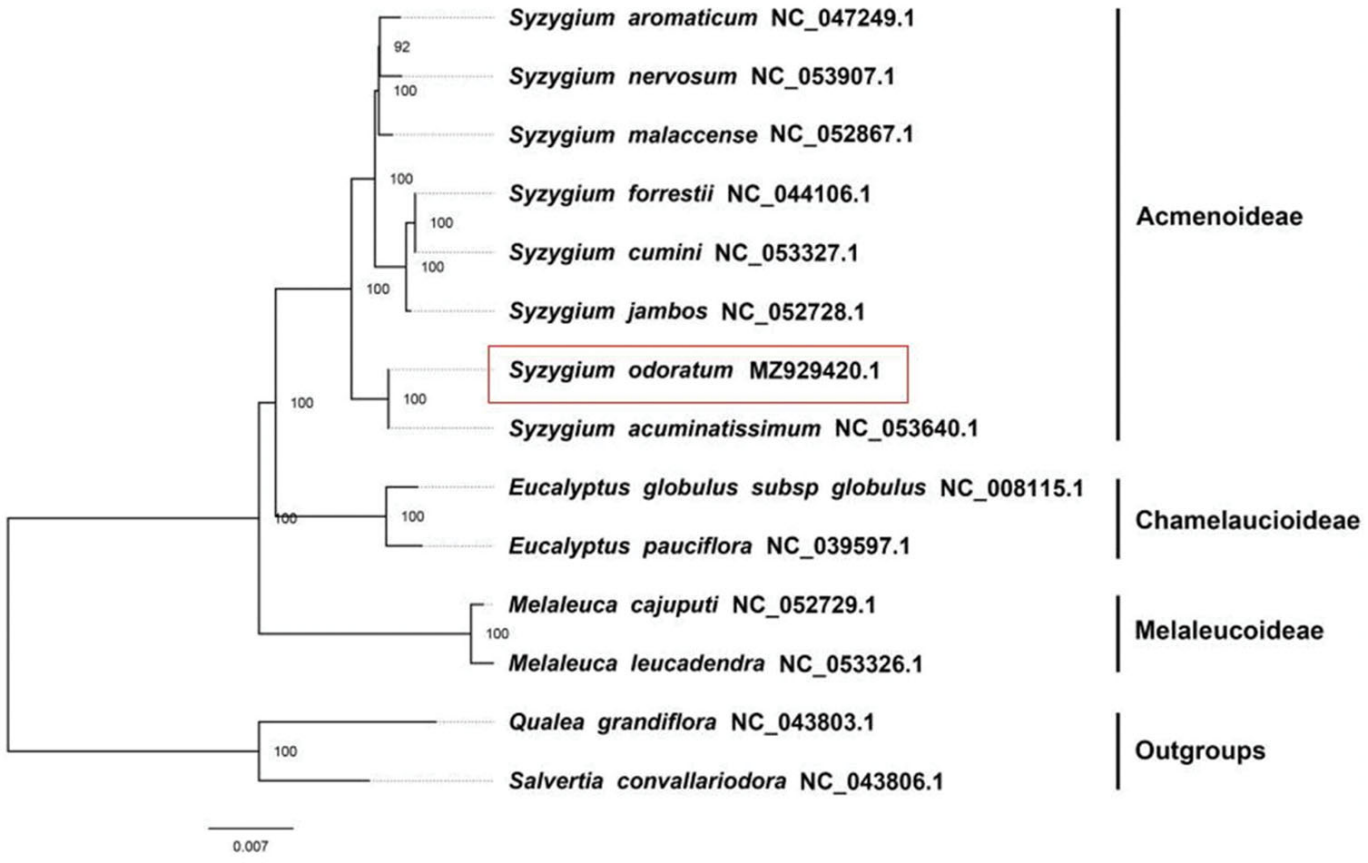
The maximum-likelihood phylogeny recovered from 14 complete plastome sequences using RAxML. The focal species of this study, *S. odoratum* (GenBank accession MZ929420.1). The box means the newly reported complete plastome of Syzygium odoratum in this study.

## Data Availability

The genome sequence data supporting the results of this study are publicly available in GenBank of NCBI (https://www.ncbi.nlm.nih.gov/) with registration number MZ929420.1. The associated BioProject, SRA, and Bio-Sample numbers are SRR15651130, PRJNA748537, and SAMN20858405, respectively.

## References

[CIT0001] Chen J. 1984. Flora of China. Vol. 53. Beijing, People’s Republic of China: Science Press; p. 94.

[CIT0002] Doyle JJ, Doyle JL. 1987. A rapid DNA isolation procedure for small quantities of fresh leaf tissue. Phytochem Bull. 19:11–15.

[CIT0003] Hahn C, Bachmann L, Chevreux B. 2013. Reconstructing mitochondrial genomes directly from genomic next-generation sequencing reads—a baiting and iterative mapping approach. Nucleic Acids Res. 41(13):e129. 23661685 10.1093/nar/gkt371 PMC3711436

[CIT0005] Stamatakis A. 2006. RAxML-VI-HPC: maximum likelihood-based phylogenetic analyses with thousands of taxa and mixed models. Bioinformatics. 22(21):2688–2690. 16928733 10.1093/bioinformatics/btl446

[CIT0006] Wyman SK, Jansen RK, Boore JL. 2004. Automatic annotation of organellar genomes with DOGMA. Bioinformatics. 20(17):3252–3255. 15180927 10.1093/bioinformatics/bth352

[CIT0008] Zhu ZX, Mu WX, Wang JH, Zhang JR, Zhao KK, Friedman CR, Wang HF. 2018. Complete plastome sequence of *Dracaena cambodiana* (Asparagaceae): a species considered “Vulnerable” in Southeast Asia. Mitochondrial DNA B Resour. 3(2):620–621. 33474263 10.1080/23802359.2018.1473740 PMC7800030

